# Safety, tolerability and immunogenicity of an active anti-Aβ_40_ vaccine (ABvac40) in patients with Alzheimer’s disease: a randomised, double-blind, placebo-controlled, phase I trial

**DOI:** 10.1186/s13195-018-0340-8

**Published:** 2018-01-29

**Authors:** Ana-María Lacosta, María Pascual-Lucas, Pedro Pesini, Diego Casabona, Virginia Pérez-Grijalba, Iván Marcos-Campos, Leticia Sarasa, Jesus Canudas, Hassnae Badi, Inmaculada Monleón, Itziar San-José, Josep Munuera, Octavio Rodríguez-Gómez, Carla Abdelnour, Asunción Lafuente, Mar Buendía, Mercè Boada, Lluis Tárraga, Agustín Ruiz, Manuel Sarasa

**Affiliations:** 1 Araclon Biotech, Vía Hispanidad 21, 50009 Zaragoza, Spain; 2 Institut de Diagnòstic per la Imatge, Hospital Universitari Germans Trias i Pujol, Badalona, Spain; 3 Memory Clinic and Research Centre, Fundació ACE Institut Català de Neurociències Aplicades, Barcelona, Spain

**Keywords:** Alzheimer’s disease, Amyloid-β, Aβ, Immunotherapy, ABvac40, Phase I

## Abstract

**Background:**

Immunotherapy targeting the amyloid-β (Aβ) peptide is a promising strategy for the treatment of Alzheimer’s disease (AD); however, none of the active or passive vaccines tested have been demonstrated to be effective to date. We have developed the first active vaccine against the C-terminal end of Aβ_40_, ABvac40, and assessed its safety and tolerability in a phase I clinical trial.

**Methods:**

A randomised, double-blind, placebo-controlled, parallel-group, phase I study of ABvac40 was conducted with patients aged 50–85 years with mild to moderate AD. Participants were entered into three separate groups according to time of study entry and were randomly allocated to receive ABvac40 or placebo (overall ratio 2:1). The first group received two half-doses of ABvac40 or placebo, whereas the second and third groups received two and three full doses, respectively. All treatments were administered subcutaneously at 4-week intervals. Patients, carers and investigators were blind to treatment allocation throughout the study. The primary objective was to assess the safety and tolerability of ABvac40 by registering all adverse events (AEs). All patients who received at least one dose of treatment were included in the safety analysis. The secondary objective was to evaluate the immunogenicity of ABvac40 by titration of specific anti-Aβ_40_ antibodies in plasma.

**Results:**

Twenty-four patients were randomly allocated: 16 patients to the ABvac40 group and 8 patients to the placebo group. All randomised patients completed the study, therefore the intention-to-treat and safety populations were identical. Overall, 71 AEs affecting 18 patients were recorded: 11 (69%) in the ABvac40 group and 7 (88%) in the placebo group (*p* = 0.6214). Neither incident vasogenic oedema nor sulcal effusion (amyloid-related imaging abnormalities corresponding to vasogenic oedema and sulcal effusions) nor microhaemorrhages (amyloid-related imaging abnormalities corresponding to microhaemorrhages and hemosiderin deposits) were detected throughout the study period in the ABvac40-treated patients. Eleven of 12 (~92%) individuals receiving three injections of ABvac40 developed specific anti-Aβ_40_ antibodies.

**Conclusions:**

ABvac40 showed a favourable safety and tolerability profile while eliciting a consistent and specific immune response. An ongoing phase II clinical trial is needed to confirm these results and to explore the clinical efficacy of ABvac40.

**Trial registration:**

ClinicalTrials.gov, NCT03113812. Retrospectively registered on 14 April 2017.

**Electronic supplementary material:**

The online version of this article (10.1186/s13195-018-0340-8) contains supplementary material, which is available to authorized users.

## Background

Alzheimer’s disease (AD) is the most common type of dementia, accounting for 50–75% of the estimated 47 million people with dementia worldwide [1]. AD is defined as a neurodegenerative disorder clinically characterised by progressive memory loss and cognitive decline. Currently, there is no effective treatment, and currently approved drugs provide only modest symptomatic benefit. Therefore, development of disease-modifying drugs is of great importance.

The amyloid cascade hypothesis of AD proposes that amyloid-β (Aβ) peptide accumulation in the brain, caused by an imbalance between Aβ production and clearance, is the initiating factor of a cascade of pathogenic events, including the formation of neurofibrillary tangles (NFTs), oxidative stress, neuroinflammation, synaptic dysfunction and neuronal loss, which eventually leads to AD dementia [2, 3]. In recent years, several active immunotherapies targeting Aβ have progressed from preclinical studies in AD mouse models to clinical trials in humans [4]; however, none of the approaches tested have shown clinical efficacy so far [5].

Several isoforms of Aβ are generated from sequential proteolytic cleavage of the amyloid precursor protein (APP), including Aβ_40_ and Aβ_42_. Aβ_40_ is the predominant variant (90%) among the secreted Aβ forms [6–8], and although Aβ_42_ is more hydrophobic and prone to aggregate, and Aβ_42_ oligomers are regarded to be the most neurotoxic species, Aβ_40_ can also produce highly toxic diffusible aggregates [9], which can be prevented in vitro by specific anti-Aβ_40_ antibodies [10]. Accordingly, researchers in several studies have proposed that a high concentration of Aβ_40_ in the brain distinguishes patients with AD from those who have senile plaques but are cognitively normal, pointing to the importance of Aβ_40_ in the onset of dementia, both in AD and in Down syndrome [11–13]. In addition, Aβ_40_ is the main component of amyloid deposition occurring in cerebral amyloid angiopathy (CAA) [14], which has a prevalence of about 80–90% in patients with AD [15]. In keeping with this, previous studies have demonstrated that specific anti-Aβ_40_ antibodies label intra- and extra-neuronal NFTs in the entorhinal cortex and the hippocampus of AD brains, and that these do not co-localise with tau NFTs, suggesting the presence of degenerating neuronal populations filled with C-terminal fragments of Aβ_x-40_ [16].

Considering all previous results suggesting that strategies targeting Aβ_40_ could represent novel disease-modifying therapies, we have developed ABvac40, the first active vaccine targeting the C-terminal end of the Aβ_40_ peptide. Unlike N-terminal end Aβ-directed antibodies, which could recognise both Aβ and their parental APP while inserted in the cell membrane, anti-C-terminal end Aβ antibodies do not bind to the unprocessed protein, preventing the accumulation of potentially toxic antigen-antibody complexes around neurons and other APP-expressing cells, which further increases the availability of circulating antibodies to interact with Aβ peptides. In addition, C-terminal (and not N-terminal) end Aβ-directed antibodies generated by ABvac40 could provide protection against N-terminally truncated and/or modified Aβ peptides, such as pyroglutamate-3 Aβ, which have been described to be highly toxic and prone to aggregation [17–21].

Therefore, the aim of this study was to assess the safety and tolerability of repeated subcutaneous administrations of an active vaccine against the C-terminal end of Aβ_40_ in patients with mild to moderate AD. In addition, we evaluated ABvac40 biological activity in terms of the immune response induced in participants by determining the plasma levels of anti-Aβ_40_ antibodies.

## Methods

A randomised, double-blind, placebo-controlled, parallel-group, single-centre, phase I study was done at the Memory Clinic and Research Center of Fundació ACE (Barcelona, Spain) to assess the safety and tolerability of repeated subcutaneous administrations of ABvac40 in patients with mild to moderate AD. The study was initiated upon approval by the independent ethics committee of the Barcelona Hospital Clinic and was conducted in accordance with the ethical and scientific principles described in the Declaration of Helsinki and International Conference on Harmonisation Guideline for Good Clinical Practice (CPMP/ICH/135/95), European guidelines for clinical trials (2001/20/CE) and Spanish legislation (Royal Decree 223/2004 of 6 February, which regulates clinical drug trials). A data and safety monitoring board of medical experts in the fields of neurology and immunology monitored the trial.

### Participants

The study population consisted of men and women aged 50–85 years with a clinical diagnosis of probable AD based on National Institute of Neurological and Communicative Diseases and Stroke/Alzheimer’s Disease and Related Disorders Association criteria and a Mini Mental State Examination score of 15–26 (mild to moderate AD). Patients were excluded if they had a history or indications of any other central nervous system disorder that could be the cause of dementia or a history or indications of cerebrovascular disease or diagnosis of possible, probable or definite vascular dementia (National Institute of Neurological Disorders and Stroke-Association Internationale pour la Recherche et l’Enseignement en Neurosciences criteria). Conventional treatments for AD were permitted if they were administered at a stable dose for at least 3 months before screening and were maintained throughout the trial. Further details of the inclusion and exclusion criteria are available in Additional file 1. All participants provided written informed consent before enrolment.

### Study design

**Fig. 1:**
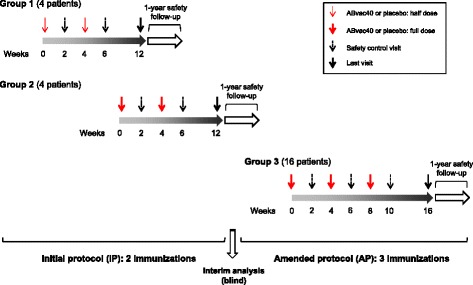
Study design

Participants were randomised into two treatment groups receiving ABvac40 or placebo. To minimise the risk associated with the use of ABvac40 in humans for the first time, a stepped recruiting protocol was followed (Fig. 1). The first four patients were randomised and treated sequentially on consecutive days with half the intended dose (two with ABvac40 and two with placebo). Once these patients successfully completed the safety control after the second injection, a second group of four patients was randomised and treated with the full dose (two with ABvac40 and two with placebo). After these eight patients, following the initial protocol (IP), had completed the safety control after the second dose, an interim analysis was carried out to monitor the immune response, maintaining the double-blinding of the study. Based on the results of this interim analysis, the protocol was amended to introduce an additional third immunisation. Thus, the remaining 16 patients (12 ABvac40 and 4 placebo) following the amended protocol (AP) received three full immunisations. On the whole, the ABvac40/placebo ratio was 2:1. The randomisation lists were prepared by an independent statistician using SAS software (SAS Institute, Cary, NC, USA). Further details about study randomisation are provided in Additional file 2.

ABvac40 and placebo treatments were dispensed in identical vials to make them indistinguishable. Only an independent representative of the sponsor worked without blinding to label the treatment kits. Patients, carers, investigators and all staff involved with the trial were blind to treatment allocation throughout the study; however, the principal investigator was permitted to unmask the treatment in case of a medical emergency.

### Procedures

In total, over the treatment period spanning 4 or 8 weeks (IP or AP, respectively), the patients received two or three administrations (IP or AP, respectively) at 4-week intervals. The vaccine was administered subcutaneously. Each dose consisted of 1 ml of ABvac40 containing 200 μg of Aβ_33–40_ peptide coupled to monomeric keyhole limpet haemocyanin suspended in the vaccine vehicle (phosphate buffer with 0.35% aluminium hydroxide in the form of Alhydrogel® [InvivoGen, San Diego, CA, USA] as adjuvant). The placebo consisted of the vaccine vehicle without the immunogenic conjugate.

Study visits were scheduled to follow a logical sequence to monitor patient safety and compliance with trial requirements. Up to 4 weeks before treatment, a screening visit and a baseline visit were carried out to ensure the suitability of the patients for the clinical trial and to define their baseline characteristics. These visits included physical and neurological examinations, blood tests, urinalysis, magnetic resonance imaging (MRI) scans, electrocardiograms (ECGs) and neuropsychological tests. MRI scans were performed in 1.5-Tesla magnets. A standard protocol was used with the following sequence: 3D T1-weighted, Alzheimer’s Disease Neuroimaging Initiative sequence; fluid-attenuated inversion recovery (FLAIR), 2D axial T2-weighted (T2W) FLAIR; T2*-weighted, axial 2D gradient echo; T2W, axial 2D spin echo; diffusion-weighted image and associated apparent diffusion coefficient map. Mesial temporal atrophy was assessed using the Scheltens scale, and detection of amyloid-related imaging abnormalities (ARIAs) was performed according to published criteria [22, 23]. As a safety measure, patients were hospitalised for drug administration at Clínica CIMA in Barcelona and kept under observation for the first 24 h. The patients were discharged from hospital only if stable and there was no reasonable suspicion of a possible allergic reaction. Two or three days later, the status of the participants was checked via a telephone interview. In addition, 2 weeks after each vaccination, the patients underwent a full safety control visit, including a control MRI scan, blood test, urinalysis and a complete physical and neurological examination. Six weeks after the last safety control visit, the final visit took place. After concluding participation in the study, patients were followed for at least 1 additional year for long-term safety control. This open-label follow-up consisted of four additional visits, which included supplementary MRI scans and blood tests.

### Outcomes

The primary objective of the study was to evaluate the safety and tolerability of multiple administrations of ABvac40 in patients with mild to moderate AD. The main variable to assess was the frequency (%) of adverse events (AEs). In this regard, special efforts were made to evaluate potential neurological AEs (cerebrovascular events, extrapyramidal symptoms, disorientation, increased gait impairment and occurrence of seizures), psychiatric AEs (hallucinations and other signs and symptoms of affective or psychotic disorders, disorientation, agitation and aggressive behaviour) and cardiovascular AEs (orthostatic hypotension, induced arrhythmias and/or increased risk of myocardial infarction). Safety assessments included the recording of all AEs, regular MRI scans, physical and neurological examinations, laboratory assessments (standard haematology, blood biochemistry and urinalysis panels), ECGs, investigator global evaluation (Clinical Global Impression of Change), assessment of vital signs and body mass index.

The secondary objective of the study was to evaluate ABvac40 biological activity in terms of the immune response induced in the participants by determining the levels of anti-Aβ_40_ antibodies in plasma, measured as the mean optical density (MOD) signal from three replicated titration enzyme-linked immunosorbent assays (ELISAs) in 96-well plates coated with the Aβ_1–40_ peptide. Antibodies bound to immobilised Aβ_1–40_ were detected with anti-human immunoglobulin G (IgG)-specific secondary antibodies coupled to horseradish peroxidase. The MOD of samples with a reported overflow by the ELISA reader was equalled to 4.08 (maximum reading value). The maximal signal increment (MSΔ) was calculated for each subject as the difference between the maximal MOD at any post-baseline visit and the MOD at baseline. To evaluate if the increment of signal was due to specific anti-Aβ_40_ antibodies, aliquoted parts of the test samples were pre-adsorbed with Aβ_33–40_ peptide and then processed by titration ELISAs in parallel with the non-pre-adsorbed samples. Patients were classified as positive responders to ABvac40 if, at a 1:10 plasma dilution, the signal increment at any post-treatment visit regarding the baseline was ≥ 3 SD and such increment was reduced in the pre-adsorbed sample by ≥ 50%. The ABvac40 biological activity in the subjects in the ABvac40 group was also expressed in antibody titres, defined as the inverse of the maximal plasma sample dilution which showed an increase in MOD ≥ 3 SD with regard to the baseline sample.

The reactivity of selected plasma samples with amyloid plaques was assessed in brain sections from 9.5-month-old APP/PS1-transgenic mice and patients with AD by immunohistochemistry as described elsewhere (plasma samples diluted 1:500 in 0.5% Triton X-100 PBS were used as the primary antibody) [24]. The ability of the antibodies raised by ABvac40 to target different forms of Aβ was analysed by immunoblotting. Briefly, Aβ_1–40_ or Aβ_1–42_ synthetic oligomers were resolved onto Tris-Tricine gels, transferred to nitrocellulose membranes and blotted with diluted plasma samples.

Additional secondary variables were assessed for exploratory purposes. The levels of Aβ peptides in plasma (Aβ_40_ and Aβ_42_) were quantified by using an Aβ ELISA kit following the manufacturer’s instructions (Araclon Biotech, Zaragoza, Spain). The levels of cytokines in plasma (interleukin [IL]-6, tumour necrosis factor-α, IL-1β, monocyte chemoattractant protein 1, IL-2 and soluble IL-2 receptor) were determined by a certified clinical analysis laboratory (Laboratorios Echevarne, Barcelona, Spain).

### Statistical analysis

Owing to the exploratory nature of the study, a formal statistical estimation of the sample size was not made. In general, categorical data were presented as counts and percentages in each category, and continuous data were reported using number of patients, mean value, SD and SEM.

**Fig. 2:**
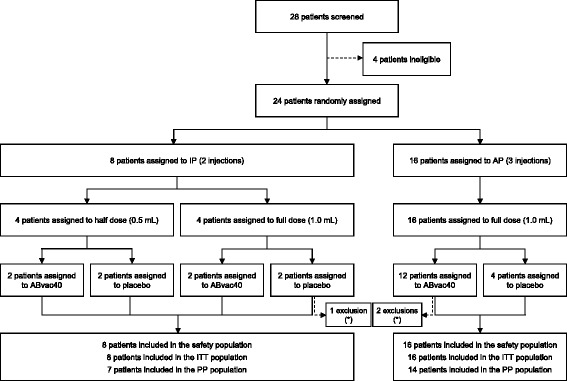
Trial profile. *IP* Initial protocol, *AP* Amended protocol, *ITT* Intention to treat, *PP* Per protocol, * Major protocol deviations

The number of AEs and the percentage of patients with AEs, overall and grouped as neurological, psychiatric and cardiovascular, were analysed and compared between the active treatment group and the control group with the chi-square test or Fisher’s exact test, as appropriate. The levels of anti-Aβ_40_ antibodies, Aβ peptides and cytokines in plasma were analysed after each visit using descriptive statistics. Statistical comparison between groups of treatment was done with the Wilcoxon rank-sum test for each time point and for the last follow-up endpoint (defined as the last observation available). The change from baseline in absolute value was analysed in an exploratory manner for each time point and for the last follow-up point. This analysis was performed separately for IP and AP patients. The significance level was set to *p* ≤ 0.05. All patients who received at least one dose of medication were included in the safety assessment (safety population and intention-to-treat [ITT] population), whereas evaluation of the biological activity was carried out in the ITT and per-protocol (PP) populations (Fig. 2). Statistical analyses were performed using SAS 9.4 software.

## Results

### Participants

Participants were recruited between 20 December 2013 and 30 March 2015. Recruitment was interrupted from 1 July 2014 to 14 January 2015 for interim analysis and submission of an amendment to the IP. A total of 28 patients were initially screened, and 24 were finally enrolled into the study. Of the enrolled patients, 16 were randomly allocated to ABvac40 treatment (2 patients received 2 half-doses, 2 patients received 2 full doses and 12 patients received 3 full doses), and 8 participants were randomly allocated to placebo (Fig. 1). All randomised patients completed the study; therefore, the safety and ITT populations were identical. However, a major protocol deviation was identified in three patients who had been treated with experimental immunotherapies in a previous clinical trial. These three patients (two in the ABvac40 group and one in the placebo group) were excluded from the PP population (Fig. 2).

Baseline patient demographics are summarised in Table 1. Briefly, the ABvac40 and placebo groups were homogeneous concerning most demographic characteristics, including distribution of *APOE* genotypes, years of education, sex and time from diagnosis; they differed only in age, with the ABvac40 group being 9.6 years older, on average, than the placebo group. All patients received a stable AD medication dose during 3 months prior to screening and throughout the study.

**Table 1:** Baseline characteristics

	Safety/ITT population (*N* = 24)	
	ABvac40 (*n* = 16)	Placebo (*n* = 8)
Age, years		
Mean (SD)	72.4 (7.2)	62.8 (6.9)
Years of education		
Mean (SD)	7.1 (3.4)	8.9 (5.4)
Sex		
Male	8 (50%)	3 (38%)
Female	8 (50%)	5 (63%)
Time from AD diagnosis, months		
Mean (SD)	18.3 (17.4)	13.0 (11.7)
*APOE* genotype		
ε3ε3	6 (38%)	3 (38%)
ε3ε4	8 (50%)	3 (38%)
ε4ε4	2 (13%)	2 (25%)
GDS		
0–10: Normal	15 (94%)	8 (100%)
11–14: Depression	1 (6%)	0 (0%)
> 14: Depression	0 (0%)	0 (0%)
Hachinski Ischemic Scale score		
< 4 Suggestive of degenerative disorder	16 (100%)	8 (100%)
4–7 Doubtful cases and mixed dementias	0 (0%)	0 (0%)
> 7 Suggestive of vascular involvement	0 (0%)	0 (0%)
Leukoaraiosis scale, total		
Mean (SD)	2.8 (2.7)	2.6 (4.1)
Microhaemorrhage presence		
Yes	4 (25%)	2 (25%)
CDR		
0.5 points	2 (13%)	4 (50%)
1 point	14 (88%)	4 (50%)
2 points	0 (0%)	0 (0%)
MMSE total score		
Mean (SD)	19.0 (2.7)	21.2 (3.4)
MMSE total score (by age and schooling)		
Mean (SD)	20.1 (2.7)	21.9 (3.3)

*Abbreviations: APOE* Apolipoprotein E, *AD* Alzheimer’s disease, *GDS* Geriatric Depression Scale, *CDR* Clinical Dementia Rating, *MMSE* Mini Mental State Examination, *ITT* Intention to treat

Data are mean (SD) or number (%)

### Safety and tolerability

The primary endpoint to assess the safety and tolerability of the study drug was the frequency of AEs. Overall, 71 AEs were recorded in 18 patients: seven out of the eight patients (88%) in the placebo group suffered at least one AE during the study, compared with 11 out of the 16 patients (69%) in the ABvac40 group (Table 2). There were no significant differences in the incidence of AEs between both groups; neither for the total number of AEs (*p* = 0.6214 for total AEs occurrence between groups) nor for these grouped as neurological, psychiatric and cardiovascular AEs (*p* = 0.2038, *p* = 1.0000 and *p* = 1.0000, respectively).

**Table 2:** Adverse events

	Safety/ITT population						
	ABvac40 (*n* = 16)		Placebo (*n* = 8)		Total (*N* = 24)		
	AEs (*n*)	No. of patients (%)	AEs (*n*)	No. of patients (%)	AEs (*n*)	No. of patients (%)	*p* Value
Total AEs	42	11 (69%)	29	7 (88%)	71	18 (75%)	0.6214
Neurological	9	5 (31%)	6	5 (63%)	15	10 (42%)	0.2038
Psychiatric	2	2 (13%)	1	1 (13%)	3	3 (13%)	1.0000
Cardiovascular	1	1 (6%)	1	1 (13%)	2	2 (8%)	1.0000

*AE* Adverse event, *ITT* Intention to treat

Analysis was done using Fisher’s exact test. *See* Additional file 3: Table S1 for a complete list of reported AEs

The most common AEs were headache, which occurred in nine individuals, and urinary tract infection, which occurred in six individuals. No other AE occurred in more than three individuals (13% of the participants). For a complete list of reported AEs and their incidence, *see* Additional file 3: Table S1. Apart from the urinary tract infections, no other relevant clinical abnormalities or changes from baseline were detected in any participant concerning haematology, blood biochemistry, ECG, vital signs, body mass index and neurological examination explored for complementary assessment of ABvac40 tolerability (data not shown). Most AEs were considered unrelated to the treatment, and only a few were considered possibly or probably related (Additional file 4: Table S2), including one clinically asymptomatic microhaemorrhage detected by MRI after the second immunisation in a patient belonging to the placebo group.

All AEs were classified as mild and did not require modification of the treatment schedule. Of particular relevance, no vasogenic oedema or sulcal effusion (amyloid-related imaging abnormalities corresponding to vasogenic oedema and sulcal effusions [ARIA-E]) was detected throughout the study period or on the four extra MRI scans of the participants taken during the additional 1-year follow-up for long-term safety control. Only one of the participants in the placebo group experienced three simultaneous serious adverse events (SAEs; hypothermia, dehydration and rhabdomyolysis) after escaping from family control and lying overnight in a dry creek. The patient was hospitalised, and the event ended 1 week afterward without sequelae.

Local reactions at the injection point occurred in 13 subjects: 9 patients in the ABvac40 group (56%) and 4 patients in the placebo group (50%). Most reactions disappeared at the safety control visit 2 weeks after the immunisation, and they were limited to redness and slight swelling, except one case followed by itching and erythema that was reported as an AE (Additional file 4: Table S2).

### Immune response

**Fig. 3:**
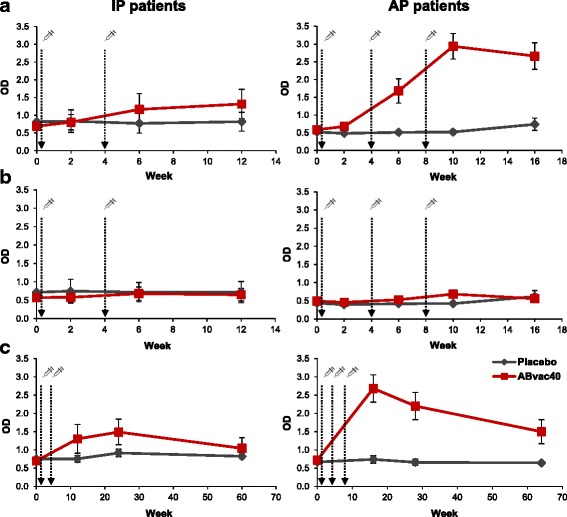
Evolution over time of the immune response of initial protocol (IP) and amended protocol (AP) patients (*left* and *right panels*, respectively) from baseline to the final visit (**a** and **b**) and during the 1-year open-label follow-up (**c**). The levels of anti-amyloid-β_40_ (Aβ_40_) antibodies in plasma are represented as the optical density (OD) in the titration enzyme-linked immunosorbent assays performed in 96-well plates coated with the Aβ_1–40_ peptide. Pre-adsorption of plasma samples with Aβ_33–40_ peptide (**b**) resulted in a reduction of > 91% of the signal compared with non-pre-adsorbed samples (**a**), suggesting that the signal corresponded to specific anti-Aβ_40_ antibodies. The levels of specific anti-Aβ_40_ antibodies remained elevated in AP patients in the ABvac40 group for up to 56 weeks after the last immunisation (**c**). Data are mean ± SEM

The assessment of the biological activity of ABvac40 was achieved by determining the plasma levels of anti-Aβ_40_ IgG antibodies. Considering the ITT population, the average MSΔ in the ABvac40 group was 1.94 (SD 1.32) optical density (OD) units (Table 3). It should be noted that the third immunisation in the AP dramatically increased the levels of anti-Aβ_40_ antibodies from an MSΔ of 0.64 (SD 0.81) OD units in the IP patients to 2.37 (SD 1.18) OD units in the subjects following the AP (Fig. 3a). Fourteen of 16 (88%) participants in the ABvac40 group were considered positive responders (3 of 4 patients following the IP and 11 of 12 patients following the AP) (Table 3). On average, > 91% of the signal registered in the native plasma samples from patients treated with ABvac40 disappeared after overnight pre-adsorption of corresponding aliquots of the same samples with the ABvac40 immunogenic peptide (Aβ_33–40_), indicating that the signal increment was due to the presence of specific anti-Aβ_40_ antibodies (Table 3 and Fig. 3b). None of the patients receiving placebo had significant specific anti-Aβ_40_ antibodies. However, it should be noted that a patient in the placebo group (S028) showed a high signal that turned out to be non-specific owing to the low percentage of signal disappearing after overnight pre-adsorption.

**Table 3:** Quantification of the immune response

Treatment	Patient	Protocol	MSΔ	SDp	MSΔ/SDp	Signal adsorbed^a^ (%)	Titres
Placebo	S002	IP	0.090	0.049	1.837	–	–
	S004	IP	0.062	0.301	0.206	–	–
	S005	IP	−0.033	0.055	−0.600	–	–
	S010	IP	0.086	0.036	2.380	–	–
	S015	AP	0.113	0.086	1.318	–	–
	S017	AP	0.055	0.065	0.841	–	–
	S020	AP	−0.076	0.118	−0.644	–	–
	S028	AP	0.872	0.065	13.421	12.72^b^	–
ABvac40	S001	IP	0.249	0.071	3.507	83.00	30
	S003	IP	0.315	0.064	4.927	96.72	10
	S008	IP	0.129	0.397	0.326	–	–
	S011	IP	1.852	0.066	28.061	90.95	810
	S012	AP	0.190	0.099	1.919	–	–
	S013	AP	3.635	0.097	37.474	95.54^c^	65,610
	S014	AP	3.093	0.104	29.737	95.52	7290
	S016	AP	2.156	0.099	21.778	95.22	270
	S018	AP	0.626	0.037	16.298	105.22	90
	S019	AP	3.526	0.099	35.616	99.63^c^	21,870
	S021	AP	2.265	0.156	14.519	87.90	270
	S022	AP	2.419	0.127	19.047	63.92	810
	S023	AP	3.461	0.029	119.345	99.25^c^	65,610
	S024	AP	1.011	0.103	9.816	72.40	90
	S025	AP	2.852	0.112	25.464	93.71	810
	S026	AP	3.230	0.104	31.058	96.81^c^	21,870

*Abbreviations: MS*Δ Maximal signal increment (in optical density), *SDp* Average SD from all visits of each patient, *IP* Initial protocol, *AP* Amended protocol, *A*β Amyloid-β

Non-responder patients are shown in bold

^a^Pre-adsorbed with 10^−4^ M Aβ_33–40_

^b^The low percentage of adsorption of this sample suggests non-specific signal

^c^Pre-adsorbed with 10^−3^ M Aβ_33–40_

**Fig. 4:**
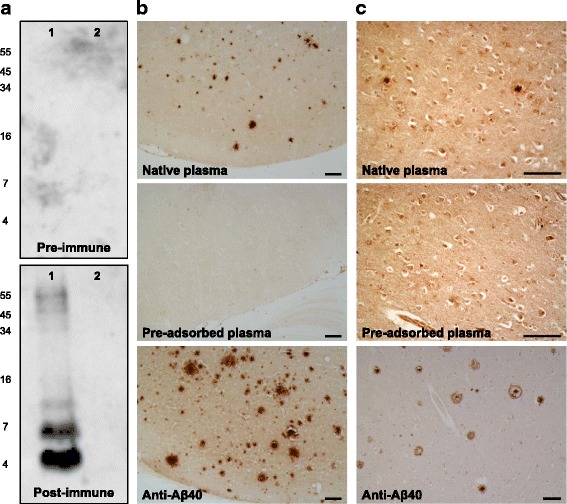
Reactivity of ABvac40-induced anti-amyloid-β (anti-Aβ) antibodies: **a** Post-immune plasma samples (week 10) from an ABvac40-treated patient (S013) recognised different forms of synthetic Aβ_40_ peptide (*lane 1*). In contrast, they did not label any form of synthetic Aβ_42_ (*lane 2*). Pre-immune plasma (week 0) did not show reactivity with Aβ_40_ or Aβ_42_. **b** and **c** Binding of plasma samples from the same patient (S013; week 10) to amyloid plaques in paraffin-embedded brain sections from APP/PS1-transgenic mice (**b**) and patients with AD (**c**). Pre-adsorption of plasma with Aβ_33–40_ peptide prevented plaque staining. Specific anti-Aβ_40_ polyclonal antibody was used as a positive control. Scale bar = 100 μm

Interestingly, the levels of specific anti-Aβ_40_ antibodies in ABvac40-treated patients in the AP subgroup remained significantly higher than pre-immune plasma levels up to 56 weeks after the last immunisation (*p* = 0.004), as observed during the 1-year follow-up for long-term safety assessment (Fig. 3c). ABvac40-induced antibodies recognised synthetic Aβ_1–40_, including monomers, dimers, trimers and oligomers; however, they did not label any form of Aβ_1–42_ (Fig. 4a). The reactivity of plasma samples from ABvac40-treated patients with amyloid brain plaques was confirmed by immunohistochemistry on brain sections from APP/PS1-transgenic mice (Fig. 4b) and patients with AD (Fig. 4c).

Regarding other exploratory secondary efficacy variables, such as the plasma levels of Aβ peptides (Additional file 5: Table S3) and cytokines (data not shown), no significant differences were found between treatment groups at the end of the clinical trial.

## Discussion

Our findings show a good safety and tolerability profile for ABvac40, because upon a relevant and specific immune response in 88% of the participants in the active arm, no SAEs were recorded in the ABvac40 group and no significant differences were found in the frequency of AEs, overall and grouped as neurological, psychiatric and cardiovascular AEs, as compared with the placebo group. All AEs detected throughout the study were classified as mild and did not require changes in treatment schedule; most of them, with the exception of mild and transient local reactions, were considered neither possibly nor probably related to the investigational medical product.

Since it was first reported that active immunisation targeting Aβ halted the progression of AD pathology in transgenic mice [25], numerous studies with promising results in animals have progressed into clinical trials. However, the first clinical trial of active immunotherapy, consisting of repeated administrations of aggregated Aβ_42_ with QS-21 as an adjuvant (AN1792), was discontinued owing to meningoencephalitis in 6% of treated patients [26]. These AEs were likely caused by an Aβ-specific T-cell-mediated Th1 immune response, which was attributed to the use of QS-21, a strong Th1-type adjuvant, and the use of full-length Aβ_1–42_ carrying T-cell-activating epitopes. Although the AN1792 clinical trial failed, long-term follow-up of responder patients showed a reduction in brain amyloid burden [27, 28] and attenuated functional decline [29–31], which supports the potential benefits of Aβ immunotherapy, provided that an Aβ-specific T-cell response can be avoided. In this regard, it is important to underline that the T-cell epitopes of the Aβ peptide have been mapped to different regions, including Aβ_1–16_ [32], Aβ_6–28_ [33] and Aβ_16–25_ [34], as well as Aβ_16–30_, Aβ_19–33_ and the Aβ_28–42_ C-terminal fragment of Aβ_42_ [35]. Nevertheless, it should be noted that those T-cell lines reactive to Aβ_28–42_ were unreactive to Aβ_1–40_, suggesting the importance of the two C-terminal amino acid residues [35]. Thus, to minimise the potential risk of T-cell responses, ABvac40 was designed using the C-terminal end of Aβ_40_ (Aβ_33–40_) and aluminium hydroxide as an adjuvant to stimulate a Th2-type immune response [36]. In line with this, no cases of meningoencephalitis were found throughout the study.

On one hand, targeting the C-terminal fragment of Aβ_40_ could have some additional safety advantages over the N-terminal, because the epitope targeted by ABvac40-elicited antibodies is concealed within the transmembrane portion of APP and therefore can be bound to antibodies only after Aβ is cleaved and secreted, avoiding cross-reactions with native APP and the apposition of antigen-antibody complexes on the neuronal cell membrane. On the other hand, after discontinuation of AN1792, passive anti-Aβ immunotherapies were favoured as a better approach to managing undesired immune responses. However, most passive immunotherapy trials have been associated with the highly frequent occurrence of ARIA [37–40], referring to a spectrum of imaging abnormalities detected on MRI scans suggestive of ARIA-E or amyloid-related imaging abnormalities corresponding to microhaemorrhages and hemosiderin deposits (ARIA-H) [41]. ARIA seem to be less frequent after active anti-Aβ immunisation [42–44]. In particular, no incidence of ARIA-E or ARIA-H was associated with ABvac40 during the study period or in the additional 1-year follow-up for long-term safety control. Researchers in a number of studies in transgenic mice have reported an increased incidence of microhaemorrhages following passive anti-Aβ_40_ immunotherapy [45, 46]. The monoclonal antibody (mAb) ponezumab, which recognises amino acids 33–40 of Aβ_40_, however, is the only passive immunotherapy that did not increase the incidence of microhaemorrhages or vasogenic oedema when administered to transgenic mice, cynomolgus monkeys or patients with mild to moderate AD [47–50].

The synthesis and kinetics of the different Aβ peptides, namely Aβ_40_ and Aβ_42_, and their differential contribution to AD physiopathology have been subject of intensive research but are not yet completely understood. Interestingly, some studies have shown that the proportion of Aβ_42_ and Aβ_40_ (the named Aβ_42_/Aβ_40_ ratio) may be more crucial for the formation of neurotoxic oligomeric conformations than the total amount of Aβ produced in the brain in the sense that changes in the Aβ_42_/Aβ_40_ ratio could favour the stabilisation of highly cytotoxic intermediate oligomers in vitro [51, 52]. These findings suggest that reducing the absolute amount of Aβ in patients with AD, such as with mAbs directed against the N-terminal end of Aβ or the central part of its sequence, could be less effective than trying to restore the appropriate Aβ_42_/Aβ_40_ ratio by specifically targeting either Aβ_42_ or Aβ_40_ by means of their C-terminal end.

Although Aβ_42_ is regarded as the most toxic species, other studies have shown that Aβ_40_ can also form cytotoxic aggregates [9, 53, 54]. Additionally, it has been observed that the levels of insoluble Aβ_40_ in the brain of patients with AD increase substantially in association with the onset of dementia [11, 12], and we have found large numbers of degenerating neurons filled with C-terminal fragments of Aβ_x-40_ (but not Aβ_x-42_) in the entorhinal cortex of AD brains [16]. These results support the idea that Aβ_40_ could play a relevant role in the pathophysiology of AD.

Moreover, along what is now described as the AD continuum, pathophysiological mechanisms other than cytotoxicity can be involved in the AD process, such as inflammation and particularly the deposition of Aβ_40_ in the cerebral blood vessels causing CAA in > 80% of patients with AD. More importantly, Aβ_40_-targeting therapies could be effective in the treatment of CAA-related inflammation (considered a naturally occurring model of ARIA) because reductions in the rate of Aβ deposition in cerebral vessels and restoration of vascular integrity have been found when anti-Aβ_40_ mAbs were administered in animal models of CAA [55].

ABvac40 was highly immunogenic because 88% of the patients receiving the vaccine showed specific anti-Aβ_40_ antibodies that recognised monomeric, oligomeric and insoluble (plaques) forms of Aβ_40_ peptide. This multi-targeted profile of the polyclonal antibodies generated by active vaccines as ABvac40 may improve their probability of success in patients at different AD pathological stages with regard to single-target mAbs. Thus, a recent phase III clinical trial with an mAb targeting soluble Aβ species (solanezumab; Expedition3 trial) has shown an inability to significantly reduce amyloid cortical burden (although a favourable tendency was apparent) in patients with mild AD [56], whereas mAbs targeting fibrillary Aβ (aducanumab) have produced very promising results [40]. However, it is also possible that the turnover of senile plaques is too slow for treatment during a relatively short period (88 weeks) with an mAb intended to cut the “supply” of soluble Aβ to cortical deposits (known to be accruing for decades before the onset of clinical symptoms), resulting in a reduction of cortical Aβ burden measurable with current neuroimaging techniques. In line with this, the solanezumab Expedition3 trial failure emphasises again the importance of confronting AD from a preventive approach, for which an active vaccine seems to be more suitable than a mAb.

As could be expected, antibody titres showed great variability owing to the individual component of the immune response. Nevertheless, it should be noted that the third immunisation, included in the AP, dramatically increased the levels of anti-Aβ_40_ antibodies with regard to the two immunisations defined in the IP while maintaining an excellent safety profile. This guarantees moving to a phase II dose-finding study to assess whether immunogenicity can be further increased more robustly across individuals. However, based on available data, no conclusions can currently be drawn about the antibody titres that could be clinically effective [57]. Interestingly, significantly elevated anti-Aβ_40_ antibody levels persisted in the ABvac40 group for up to 56 weeks after the last immunisation in those patients following the AP, which could offer long-term advantages owing to the continuous production of potentially therapeutic antibodies over time, contributing to the expected benefits of active immunisation as a cost-effective and long-term therapeutic strategy for AD [58].

Besides this, the present study has some limitations intrinsic to this initial stage of development. Because this first-in-human administration of ABvac40 was intended primarily to assess safety and tolerability, we only enrolled a limited number of patients (with unknown amyloid status) from only one centre in one country, and consequently the study was not powered to detect low-incidence AEs or changes in disease biomarkers. Therefore, we considered that in these conditions it was not worthwhile to expose patients to invasive procedures required for the assessment of amyloid biomarkers; nevertheless, these crucial measurements will be approached in an adequately powered phase II trial.

## Conclusions

Previous evidence suggests that Aβ_40_ could have an essential role in AD. Accordingly, in the present work, we have assessed the safety and tolerability of ABvac40, a novel active vaccine against the C-terminal end of Aβ_40_, in patients with mild to moderate AD. This first-in-class study has shown that ABvac40 elicited a consistent and specific immune response against the C-terminal end of Aβ_40_ while maintaining a favourable safety and tolerability profile. These results show that active immunisation is a safe therapeutic strategy for AD and also that the C-terminal end of Aβ_40_ is a promising epitope to be considered in immunotherapy approaches, pointing to ABvac40 as a promising candidate for the treatment of AD. Additional studies including larger cohorts and longer follow-up are warranted to confirm safety assessments and to establish the therapeutic range and clinical efficacy of ABvac40.

## Additional files

Additional file 1: Inclusion and exclusion criteria (detailed). (DOCX 16 kb)

Additional file 2 Randomisation (detailed). (DOCX 13 kb)

Additional file 3: Table S1. Frequency of adverse events (detailed). (DOCX 26 kb)

Additional file 4: Table S2. Relationship of adverse events (AEs) with the treatment. (DOCX 15 kb)

Additional file 5: Table S3. Quantification of Aβ_40_ and Aβ_42_ levels in plasma. (DOCX 14 kb)

## Abbreviations

AD: Alzheimer’s disease
AE: Adverse event
AP: Amended protocol
*APOE*: Apolipoprotein E
APP: Amyloid precursor protein
ARIA: Amyloid-related imaging abnormalities
ARIA-E: Amyloid-related imaging abnormalities corresponding to vasogenic oedema and sulcal effusions
ARIA-H: Amyloid-related imaging abnormalities corresponding to microhaemorrhages and hemosiderin deposits
Aβ: Amyloid-β
CAA: Cerebral amyloid angiopathy
CDR: Clinical Dementia Rating
ECG: Electrocardiogram
ELISA: Enzyme-linked immunosorbent assay
FLAIR: Fluid-attenuated inversion recovery
GDS: Geriatric Depression Scale
IgG: Immunoglobulin G
IL: Interleukin
IP: Initial protocol
ITT: Intention to treat
mAb: Monoclonal antibody
MMSE: Mini Mental State Examination
MOD: Mean optical density
MRI: Magnetic resonance imaging
MSΔ: Maximal signal increment
NFT: Neurofibrillary tangle
OD: Optical density
PP: Per protocol
SAE: Serious adverse event
T2W: T2-weighted
