# Supplementary material for: Safety, tolerability and immunogenicity of an active anti-Aβ40 vaccine (ABvac40) in patients with Alzheimer’s disease: a randomised, double-blind, placebo-controlled, phase I trial
Source: Alzheimers Res Ther. 2018 Jan 29;10:12. doi: 10.1186/s13195-018-0340-8 (PMC5789644; doi:10.1186/s13195-018-0340-8)
Supplement: Supplementary file 1 — Inclusion and exclusion criteria (detailed). (DOCX 16 kb) [file 13195_2018_340_MOESM1_ESM.docx]

**Inclusion & Exclusion criteria (detailed)**

Inclusion criteria

The subjects had to meet the following criteria upon enrolment:

1. Clinical diagnosis suggesting Alzheimer’s disease (AD) based on the criteria of the National Institute of Neurological and Communicative Diseases and Stroke/Alzheimer’s Disease and Related Disorders Association (NINCDS-ADRDA).

2. The results of the patient’s MRI brain scan had to be concordant with the diagnosis of AD according to the following criteria: Scheltens scale, and quantitative measurement of white matter and past hemorrhages.

3. Severity of Alzheimer’s disease (AD) assessed as mild or moderate using the Mini Mental State Examination (MMSE) scale. Mild or moderate AD was confirmed if the MMSE score varied between 15 and 26.

4. The Hachinski ischemic scale was used to distinguish AD from multi-infarct dementia. A score of ≤4 suggested AD.

5. Score on the Clinical Dementia Rating Scale (CDR) of 0.5 or 1.0.

6. Be receiving a stable dose of treatment for AD for three months before the selection visit, and expecting to continue treatment for the duration of the study (if appropriate).

7. Stable treatment for other illnesses may be administered 30 days prior to V0 (selection).

8. Males or females from 50 to 85 years of age, inclusive (at the time of signing informed consent).

9. The patient (or a close relative or legal representative) should read the patient information sheet, agreed to participate in the clinical trial and signed IC (the patient and a close relative or legal representative).

10. Presence of a stable caregiver willing to attend patient visits during the study.

11. Sufficient visual and auditory capacity to undertake the neuropsychological tests.

12. Positive assessment of the candidate by the investigator for complying with the requirements and procedures of the study.

Exclusion criteria

Subjects presenting any of the following criteria upon enrolment were not included into the study:

1. Pregnant women or women of child-bearing age.

2. Patients whose general state of health was such that it did not allow completion of the trial or that made taking part in the trial difficult, as judged by the investigator.

3. Participation in another clinical trial in the 3 months prior to visit 0 or during the 12 months prior to the selection visit in the case of patients who had participated in trials where the study drug was aimed at modifying the progression of AD.

4. Known allergy to the vaccine components or history of anaphylaxis, severe allergic reaction or history of hypersensitivity to any of the components of the formulation. Allergy to fish or shellfish.

5. Absolute (having a pacemaker or implantable defibrillator) or relative (bare metal stent or stent implanted in the last 6 months) contraindications to MRI examination.

6. Surgery (with general anesthetic) during the 3 months prior to admission into the study and/or surgery planned at any point during the study period.

7. History or presence of autoimmune disease.

8. Presence or history of immunodeficiency (e.g. HIV).

9. Recent history of cancer (≤3 years since the last specific treatment). (Exceptions: basal-cell carcinoma, intraepithelial cervical neoplasia).

10. Active infectious disease (e.g. hepatitis B, C) or history of chronic viral hepatitis or other chronic hepatic disorders.

11. Major systemic condition (e.g. chronic renal failure, chronic hepatopathy, uncontrolled diabetes, uncontrolled congestive heart failure, other deficiencies).

12. History of asthma or reactive airway disease presenting as bronchospasm in the last 6 months or currently on a regular anti-asthmatic drug treatment.

13. Poorly controlled diseases like poorly controlled hypertension (systolic arterial tension >160 mmHg or diastolic arterial tension >100 mmHg, as an average of 3 measurements) or poorly controlled diabetes according to the investigator’s opinion (HbA1c > 12.0).

14. Significant alterations in hematological, biochemical or urine analytical parameters, particularly those relating to levels of vitamin B12, folic acid or thyroid tests, and including the possibility of clinically significant anemia.

15. Hypothyroidism, defined as any major alteration in thyrotropin (TSH). Patients with corrected hypothyroidism could participate in the study providing that treatment had remained stable for three months immediately prior to admission to the study, and provided that it was ruled out as the cause of or as contributing to the severity of the subject’s dementia.

16. Positive serology for syphilis (except where neurosyphilis had previously been ruled out).

17. History of major psychiatric illness such as schizophrenia, bipolar disorder or major depressive disorder during the last year (according to DSM-IV criteria).

18. Metabolic or toxic encephalopathy or dementia due to a general medical illness.

19. Abuse or excessive consumption of alcohol or drugs according to the DSM-IV criteria.

20. Wernicke’s encephalopathy.

21. History or indications of any other CNS disorder that could be the cause of the dementia (demyelinating, or infectious or inflammatory CNS disease, Creutzfeldt-Jakob disease, Parkinson’s disease, Huntington’s disease, brain tumor, subdural hematoma, etc.).

22. History or indications of cerebrovascular disease (ischemic or hemorrhagic ictus, transient ischemic attack), or diagnosis of possible, probable or definite vascular dementia according to NINDS-AIREN criteria.

23. History of intracerebral hemorrhage caused by any of the following: cerebral amyloid angiopathy, uncontrolled hypertension, cerebral arteriovenous malformation, coagulopathy, CNS vasculitis or any other pathology considered by the investigator and/or medical monitor to be a risk factor for intracerebral hemorrhage.

24. Prior or current treatment with experimental immunotherapies, including IVIG or vaccines against AD.

25. Treatment with systemic corticosteroids or other immunosuppressants during the 30 days prior to visit 0.

26. Change in treatments doses for AD or hypothyroidism during the 3 months prior to visit 0.

27. Change in dose of drugs for concurrent illnesses appearing in the patient’s clinical history during the 30 days prior to visit 1, if they were clinically relevant.

28. Patients who had been previously included in the trial, except those patients who failed the screening for reasons that had since been resolved, and so their re-entry into the trial could be considered. Those who received their randomization number and then left the trial remained excluded.
