# Supplementary material for: Safety, tolerability and immunogenicity of an active anti-Aβ40 vaccine (ABvac40) in patients with Alzheimer’s disease: a randomised, double-blind, placebo-controlled, phase I trial
Source: Alzheimers Res Ther. 2018 Jan 29;10:12. doi: 10.1186/s13195-018-0340-8 (PMC5789644; doi:10.1186/s13195-018-0340-8)
Supplement: Supplementary file 2 — Randomisation (detailed). (DOCX 13 kb) [file 13195_2018_340_MOESM2_ESM.docx]

**Randomisation (detailed)**

An independent statistician not involved in the study prepared two separate randomisation lists, and sent one without treatment details to the principal investigator and one with treatment details to the Independent Representative of the Sponsor (IRS) for the packaging and labelling of the study medication. The IRS prepared three sets of opaque and blind emergency envelopes, containing information of the treatment received for each patient.

Randomisation was done using a SAS program so that the first eight patients were randomized in one block of eight elements in a 1:1 ABvac40-to-placebo ratio, whereas the other 16 patients were randomised in four blocks of four elements in a 2:1 ratio. Randomisation numbers were consecutive, starting with R-01, whereas selection numbers, also chosen consecutively, had three digits preceded by the letter ‘S-’.

The responsible for the study at the center distributed the vaccination kits following the consecutive numeration, so that the first suitable patient received the vaccination kit R-01 and the last suitable patient received vaccination kit R-24.
