# Supplementary material for: Safety, tolerability and immunogenicity of an active anti-Aβ40 vaccine (ABvac40) in patients with Alzheimer’s disease: a randomised, double-blind, placebo-controlled, phase I trial
Source: Alzheimers Res Ther. 2018 Jan 29;10:12. doi: 10.1186/s13195-018-0340-8 (PMC5789644; doi:10.1186/s13195-018-0340-8)
Supplement: Supplementary file 3 — Frequency of adverse events (detailed). (DOCX 26 kb) [file 13195_2018_340_MOESM3_ESM.docx]

**Table S1: Frequency of adverse events (detailed)**

|  | **Safety / ITT population** | | | | | |
| --- | --- | --- | --- | --- | --- | --- |
|  | **ABvac40 (N=16)** | | **Placebo (N=8)** | | **Total (N=24)** | |
|  | **# Patients** | **# AEs** | **# Patients** | **# AEs** | **# Patients** | **# AEs** |
| **Cardiac disorders** | 1 (6%) | 1 (2%) | 0 (0%) | 0 (0%) | 1 (4%) | 1 (1%) |
| **Bradycardia** | 1 (6%) | 1 (2%) | 0 (0%) | 0 (0%) | 1 (4%) | 1 (1%) |
| **Eye disorders** | 0 (0%) | 0 (0%) | 1 (13%) | 1 (3%) | 1 (4%) | 1 (1%) |
| **Eye pruritus** | 0 (0%) | 0 (0%) | 1 (13%) | 1 (3%) | 1 (4%) | 1 (1%) |
| **Gastrointestinal disorders** | 1 (6%) | 4 (10%) | 2 (25%) | 2 (7%) | 3 (13%) | 6 (8%) |
| **Diarrhoea** | 1 (6%) | 1 (2%) | 0 (0%) | 0 (0%) | 1 (4%) | 1 (1%) |
| **Oral leukoedema** | 0 (0%) | 0 (0%) | 1 (13%) | 1 (3%) | 1 (4%) | 1 (1%) |
| **Vomiting** | 1 (6%) | 3 (7%) | 1 (13%) | 1 (3%) | 2 (8%) | 4 (6%) |
| **General disorders and administration site conditions** | 6 (38%) | 6 (14%) | 3 (38%) | 3 (10%) | 9 (38%) | 9 (13%) |
| **Application site eczema** | 1 (6%) | 1 (2%) | 0 (0%) | 0 (0%) | 1 (4%) | 1 (1%) |
| **General physical health deterioration** | 1 (6%) | 1 (2%) | 0 (0%) | 0 (0%) | 1 (4%) | 1 (1%) |
| **Hypothermia** | 0 (0%) | 0 (0%) | 1 (13%) | 1 (3%) | 1 (4%) | 1 (1%) |
| **Inflammation** | 0 (0%) | 0 (0%) | 1 (13%) | 1 (3%) | 1 (4%) | 1 (1%) |
| **Injection site swelling** | 1 (6%) | 1 (2%) | 0 (0%) | 0 (0%) | 1 (4%) | 1 (1%) |
| **Oedema peripheral** | 1 (6%) | 1 (2%) | 0 (0%) | 0 (0%) | 1 (4%) | 1 (1%) |
| **Pain** | 1 (6%) | 1 (2%) | 0 (0%) | 0 (0%) | 1 (4%) | 1 (1%) |
| **Pyrexia** | 1 (6%) | 1 (2%) | 1 (13%) | 1 (3%) | 2 (8%) | 2 (3%) |
| **Infections and infestations** | 5 (31%) | 7 (17%) | 4 (50%) | 6 (21%) | 9 (38%) | 13 (18%) |
| **Gingival abscess** | 0 (0%) | 0 (0%) | 1 (13%) | 1 (3%) | 1 (4%) | 1 (1%) |
| **Nasopharyngitis** | 1 (6%) | 1 (2%) | 1 (13%) | 1 (3%) | 2 (8%) | 2 (3%) |
| **Papilloma viral infection** | 0 (0%) | 0 (0%) | 1 (13%) | 1 (3%) | 1 (4%) | 1 (1%) |
| **Urinary tract infection** | 4 (25%) | 5 (12%) | 2 (25%) | 3 (10%) | 6 (25%) | 8 (11%) |
| **Viral pharyngitis** | 1 (6%) | 1 (2%) | 0 (0%) | 0 (0%) | 1 (4%) | 1 (1%) |
| **Injury, poisoning and procedural complications** | 1 (6%) | 2 (5%) | 2 (25%) | 2 (7%) | 3 (13%) | 4 (6%) |
| **Contusion** | 1 (6%) | 2 (5%) | 0 (0%) | 0 (0%) | 1 (4%) | 2 (3%) |
| **Rib fracture** | 0 (0%) | 0 (0%) | 1 (13%) | 1 (3%) | 1 (4%) | 1 (1%) |
| **Subdural haematoma** | 0 (0%) | 0 (0%) | 1 (13%) | 1 (3%) | 1 (4%) | 1 (1%) |
| **Metabolism and nutrition disorders** | 1 (6%) | 1 (2%) | 2 (25%) | 2 (7%) | 3 (13%) | 3 (4%) |
| **Decreased appetit** | 1 (6%) | 1 (2%) | 0 (0%) | 0 (0%) | 1 (4%) | 1 (1%) |
| **Dehydration** | 0 (0%) | 0 (0%) | 1 (13%) | 1 (3%) | 1 (4%) | 1 (1%) |
| **Hypercholesterolaemia** | 0 (0%) | 0 (0%) | 1 (13%) | 1 (3%) | 1 (4%) | 1 (1%) |
| **Musculoskeletal and connective tissue disorders** | 3 (19%) | 4 (10%) | 1 (13%) | 1 (3%) | 4 (17%) | 5 (7%) |
| **Back pain** | 3 (19%) | 3 (7%) | 0 (0%) | 0 (0%) | 3 (13%) | 3 (4%) |
| **Neck pain** | 1 (6%) | 1 (2%) | 0 (0%) | 0 (0%) | 1 (4%) | 1 (1%) |
| **Rhabdomyolysis** | 0 (0%) | 0 (0%) | 1 (13%) | 1 (3%) | 1 (4%) | 1 (1%) |
| **Nervous system disorder** | 5 (31%) | 9 (21%) | 5 (63%) | 6 (21%) | 10 (42%) | 15 (21%) |
| **Dizziness** | 2 (13%) | 2 (5%) | 1 (13%) | 1 (3%) | 3 (13%) | 3 (4%) |
| **Headache** | 4 (25%) | 7 (17%) | 5 (63%) | 5 (17%) | 9 (38%) | 12 (17%) |
| **Psychiatric disorders** | 2 (13%) | 2 (5%) | 1 (13%) | 1 (3%) | 3 (13%) | 3 (4%) |
| **Anxiety** | 1 (6%) | 1 (2%) | 0 (0%) | 0 (0%) | 1 (4%) | 1 (1%) |
| **Disorientation** | 1 (6%) | 1 (2%) | 0 (0%) | 0 (0%) | 1 (4%) | 1 (1%) |
| **Insomnia** | 0 (0%) | 0 (0%) | 1 (13%) | 1 (3%) | 1 (4%) | 1 (1%) |
| **Respiratory, thoracic and mediastinal disorders** | 2 (13%) | 4 (10%) | 2 (25%) | 3 (10%) | 4 (17%) | 7 (10%) |
| **Asthmatic crisis** | 1 (6%) | 1 (2%) | 0 (0%) | 0 (0%) | 1 (4%) | 1 (1.%) |
| **Cough** | 1 (6%) | 1 (2%) | 1 (13%) | 1 (3%) | 2 (8%) | 2 (3%) |
| **Dysphonia** | 1 (6%) | 1 (2%) | 0 (0%) | 0 (0%) | 1 (4%) | 1 (1%) |
| **Dyspnoea** | 1 (6%) | 1 (2%) | 0 (0%) | 0 (0%) | 1 (4%) | 1 (1%) |
| **Nasal congestion** | 0 (0%) | 0 (0%) | 1 (13%) | 1 (3%) | 1 (4%) | 1 (1%) |
| **Sneezing** | 0 (0%) | 0 (0%) | 1 (13%) | 1 (3%) | 1 (4%) | 1 (1%) |
| **Skin and subcutaneous tissue disorders** | 1 (6%) | 2 (5%) | 0 (0%) | 0 (0%) | 1 (4%) | 2 (3%) |
| **Erythema** | 1 (6%) | 1 (2%) | 0 (0%) | 0 (0%) | 1 (4%) | 1 (1%) |
| **Rash vesicular** | 1 (6%) | 1 (2%) | 0 (0%) | 0 (0%) | 1 (4%) | 1 (1%) |
| **Surgical and medical procedures** | 0 (0%) | 0 (0%) | 1 (13%) | 1 (3%) | 1 (4%) | 1 (1%) |
| **Limb operation** | 0 (0%) | 0 (0%) | 1 (13%) | 1 (3%) | 1 (4%) | 1 (1%) |
| **Vascular disorders** | 0 (0%) | 0 (0%) | 1 (13%) | 1 (3%) | 1 (4%) | 1 (1%) |
| **Microhaemorrhage** | 0 (0%) | 0 (0%) | 1 (13%) | 1 (3%) | 1 (4%) | 1 (1%) |
