# Supplementary material for: Safety, tolerability and immunogenicity of an active anti-Aβ40 vaccine (ABvac40) in patients with Alzheimer’s disease: a randomised, double-blind, placebo-controlled, phase I trial
Source: Alzheimers Res Ther. 2018 Jan 29;10:12. doi: 10.1186/s13195-018-0340-8 (PMC5789644; doi:10.1186/s13195-018-0340-8)
Supplement: Supplementary file 4 — Relationship of adverse events (AEs) with the treatment. (DOCX 15 kb) [file 13195_2018_340_MOESM4_ESM.docx]

**Table S2: Relation of adverse events (AEs) with the treatment**

|  | | **Safety / ITT population** | | |
| --- | --- | --- | --- | --- |
|  | **ABvac40 (N=16)** | | **Placebo (N=8)** | **Total (N=24)** |
| **Possible** |  | |  |  |
| **Number of patients with at least one AE** | 2 (13%) | | 1 (13%) | 3 (13%) |
| **General physical health deterioration** | 1 (6%) | | 0 (0%) | 1 (4%) |
| **Injection site swelling** | 1 (6%) | | 0 (0%) | 1 (4%) |
| **Headache** | 1 (6%) | | 0 (0%) | 1 (4%) |
| **Microhaemorrhage** | 0 (0%) | | 1 (13%) | 1 (4%) |
| **Probable** |  | |  |  |
| **Number of patients with at least one AE** | 1 (6%) | | 0 (0%) | 1 (4%) |
| **Erythema** | 1 (6%) | | 0 (0%) | 1 (4%) |
