# Supplementary material for: Safety, tolerability and immunogenicity of an active anti-Aβ40 vaccine (ABvac40) in patients with Alzheimer’s disease: a randomised, double-blind, placebo-controlled, phase I trial
Source: Alzheimers Res Ther. 2018 Jan 29;10:12. doi: 10.1186/s13195-018-0340-8 (PMC5789644; doi:10.1186/s13195-018-0340-8)
Supplement: Supplementary file 5 — Quantification of Aβ40 and Aβ42 levels in plasma. (DOCX 14 kb) [file 13195_2018_340_MOESM5_ESM.docx]

**Table S3: Quantification of Aβ40 and Aβ42 levels in plasma**

|  |  |  |  |  |  |  |  |  |
| --- | --- | --- | --- | --- | --- | --- | --- | --- |
|  | **Safety / ITT population** | | | | | | | |
|  | **IP patients (N=8)** | | | | **AP patients (N=16)** | | | |
|  | **Aβ40 (pg/ml)** | | **Aβ42 (pg/ml)** | | **Aβ40 (pg/ml)** | | **Aβ42 (pg/ml)** | |
|  | **Baseline** | **Final** | **Baseline** | **Final** | **Baseline** | **Final** | **Baseline** | **Final** |
| **Placebo** | 282.5  (26.2) | 247.4  (79.7) | 22.4  (11.0) | 28.0  (4.8) | 225.1  (75.7) | 239.3  (20.0) | 19.8  (5.8) | 18.3  (6.8) |
| **ABvac40** | 269.4  (20.0) | 273.8  (28.6) | 40.5  (20.2) | 20.6  (17.1) | 306.6  (44.0) | 256.8  (46.2) | 25.1  (7.7) | 20.6  (7.1) |
| **p value** | **0.486** | **1.000** | **0.114** | **0.400** | **0.020** | **0.212** | **0.316** | **0.661** |

Levels of Aβ40 and Aβ42 in plasma were quantified by an Aβ ELISA kit following the manufacturer instructions (Araclon Biotech, Zaragoza, Spain). The analysis was performed separately for IP patients and for AP patients. Data are mean (SD).

Levels of Aβ40 were significantly higher in the ABvac40 group than in the placebo group in AP patients at baseline (possibly as a consequence of significantly different ages, as shown in Table 1); however, no significant treatment effect was found on the levels of any of the peptides. Intrinsic variability of the assay and among the biological samples, and the limited number of patients preclude any conclusion on this regard.
